# Natural Sulfur Compounds in Mineral Waters: Implications for Human Health and Disease

**DOI:** 10.3390/ijms262110807

**Published:** 2025-11-06

**Authors:** Mauro Vaccarezza, Marco Vitale, Paola Falletta, Orsola di Martino

**Affiliations:** 1 Curtin Medical School, Faculty of Health Sciences, Curtin University, Bentley, Perth, WA 6845, Australia; mauro.vaccarezza@curtin.edu.au; 2 Curtin Medical Research Institute (Curtin-MRI), Faculty of Health Sciences, Curtin University, Bentley, Perth, WA 6845, Australia; 3 Department of Environmental Science and Prevention, University of Ferrara, 43121 Ferrara, Italy; 4 Faculty of Medicine and Surgery, Vita-Salute University—San Raffaele, 20132 Milan, Italy; 5 Foundation for Scientific Research in Balneology (FoRST), 00198 Rome, Italy; 6 Department of Medicine and Surgery (DiMeC), University of Parma, 43100 Parma, Italy; orsola.dimartino@unipr.it

**Keywords:** spa therapy, balneotherapy, thermal medicine, sulfur compounds, mineral water, spa water

## Abstract

Natural sulfur compounds found in various mineral spring waters have attracted considerable interest due to their possible health benefits and healing qualities. Key substances such as hydrogen sulfide (H_2_S), sulfate (SO_4_^2−^), and thiosulfate (S_2_O_3_^2−^) are essential to numerous physiological functions. This overview delves into the biochemical pathways through which these sulfur compounds exert their influence, emphasizing their roles as antioxidants, anti-inflammatories, and detoxifying agents. Furthermore, it investigates the therapeutic promise of mineral waters rich in sulfur for various diseases like arthritis, skin ailments, and heart diseases. Emerging studies indicate that regular use or topical application of these waters could enhance health outcomes and aid in the prevention of a multitude of diseases. Nonetheless, additional research is required to clarify sulfur water’s mechanisms of action and to develop standardized protocols for their therapeutic applications. This descriptive review highlights the significance of integrating natural sulfur compounds into comprehensive health strategies and advocates for ongoing investigation into their advantages in medical contexts.

## 1. Introduction

In recent years, there has been a remarkable and growing interest in the world of natural sulfur compounds, particularly those found in the therapeutic and rejuvenating mineral waters that emerge from the earth’s geothermal springs. This heightened interest has been fueled by the various health benefits and unique therapeutic properties that these compounds possess, which have attracted considerable attention from both researchers and health providers alike. Sulfur compounds play crucial roles in numerous biological processes, primarily due to their significant involvement in metabolic pathways and their function as vital signaling molecules within the body [1,2,3,4,5]. Water that is rich in these beneficial sulfur compounds, often sourced from geothermal springs, mineral-rich regions, or specific salt deposits, offers significant potential for a wide range of therapeutic applications. These applications include everything from providing anti-inflammatory effects to exhibiting antioxidative properties that may improve health conditions and aid in the treatment of various diseases [6,7]. The purpose of this literature review is to synthesize the current understanding of natural sulfur compounds in mineral waters, focusing intently on their implications for human health and disease, all based on robust scientific evidence gathered through extensive research. Through this exploration, we aim to illuminate the rich landscape of natural sulfur compounds and their promising role in helping public health systems to enhance people’s health conditions, essentially by addressing chronicity.

## 2. Properties of Natural Sulfur Compounds

Sulfur-based compounds can be found in both oxidized and reduced forms. Oxidized sulfur compounds achieve a higher oxidation state, whereas reduced sulfur compounds are converted to a lower oxidation state. The distinction between oxidized and reduced forms of sulfur is critical to understand the metabolic processes and environmental functions to which they are associated. Natural sulfur compounds can exist in a fascinating variety of forms, including sulfides, sulfates, and elemental sulfur, each possessing its own unique and distinct chemical properties that can significantly influence human physiology in various ways. For example, hydrogen sulfide (H_2_S), which is a common sulfur compound found abundantly in mineral-rich waters, has been notably recognized for its profound and significant anti-inflammatory effects. For example, at the Roman Baths in Bath, a classic Roman site still in use, water is sulfate-rich (~1015–1080 mg/L SO_4_^2−^) with substantial calcium (Ca^2+^), chlorine (Cl^−^), sodium (Na^+^) and hydrogen carbonate (HCO_3_^−^); it is not a strongly “sulfurous” (H_2_S-rich) spring. In Italy, several long-standing spas show higher sulfide concentrations: at Saturnia, H_2_S is ~14–14.5 mg/L with sulfate ~1500 mg/L, while Tobiano waters used for inhalation are classified as “strong” (>100 mg/L H_2_S). These values provide realistic typical and upper bounds for H_2_S in natural waters.

Extensive studies have demonstrated that H_2_S can effectively inhibit inflammatory responses in the body, particularly by interfering with the nuclear factor-kB (NF-kB) signaling pathway, a crucial mechanism involved in the regulation of immune responses. This remarkable ability has been illustrated in numerous fibroblast studies, highlighting the potential therapeutic benefits of H_2_S in treating conditions associated with chronic inflammation and promoting overall health [8,9]. Other sulfur-containing minerals, such as those derived from natural sources like certain types of volcanic rocks and mineral springs, have similarly been associated with significant reductions in inflammation and effective pain relief. This growing body of evidence supports their therapeutic use in various health conditions, including chronic skin diseases like eczema and psoriasis [10], as well as arthritis, where joint pain and swelling are prevalent. These minerals are believed to act by enhancing the body’s natural healing processes, promoting cellular repair, and ultimately providing relief to individuals suffering from these debilitating ailments [6,7,8,9,10,11,12,13,14,15,16,17,18]. Of note, in addition to a large literature reporting positive effects in cell lines and animal models, evidence of efficacy in several clinical trials has been reported [17,19,20,21,22,23,24,25,26,27,28,29,30,31,32,33,34,35,36].

## 3. Role of Hydrogen Sulfide in Human Pathophysiology

Interestingly, the earliest insights into the role of H_2_S in human biology emerged not from physiological studies, but from toxicological investigations [37]. Cases of H_2_S poisoning revealed that survivors often suffer from cognitive impairments, prompting researchers to explore the neurotoxic effects of this gas. Experimental studies in animal models have shown that exposure to H_2_S leads to significant alterations in neurotransmitter levels, underscoring the brain’s particular vulnerability to H_2_S toxicity. In a pivotal study, Warenycia and colleagues measured H_2_S accumulation in the brains of rats exposed to the gas and found that detectable levels persisted even in the absence of ongoing exposure [38]. Although these concentrations may have been overestimated due to methodological limitations, the findings nonetheless confirmed the presence of endogenously produced H_2_S in the brain [39].

**Figure 1:**
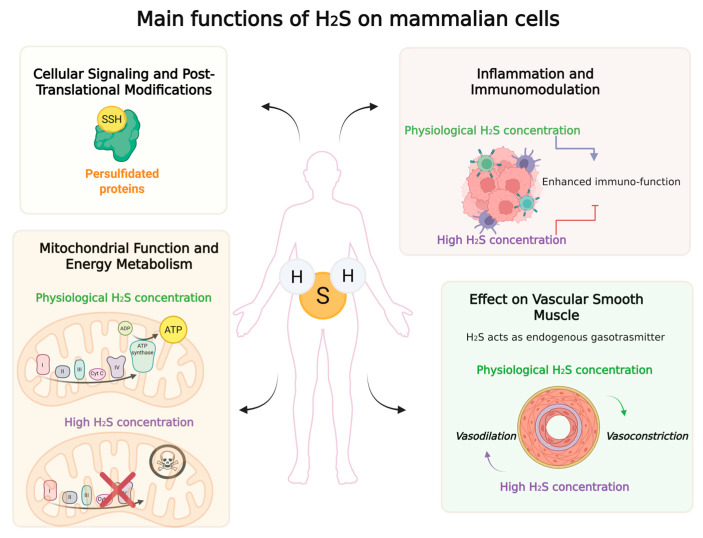
Main functions of H_2_S in mammalian cells.

Building on this discovery, Snyder and his team introduced the concept of S-sulfuration (commonly refereed as persulfidation or also S-sulfhydration)—a post-translational modification in which H_2_S modulates the function of specific proteins by modifying cysteine residues [40]. However, this mechanism has since been refined to emphasize the role of hydrogen polysulfides (H_2_Sₙ) rather than H_2_S itself, particularly in the context of protein modification. Notably, H_2_S has been shown to target oxidized cysteine residues, including those that are S-nitrosylated or S-sulfenylated, thereby influencing redox signaling pathways [41]. At the molecular level, the biosynthesis of H_2_S in mammals is orchestrated by a trio of well-characterized enzymes: cystathionine β-synthase (CBS), cystathionine γ-lyase (CSE), and 3-mercaptopyruvate sulfurtransferase (3-MST) [1,42,43]. CBS and CSE are pyridoxal phosphate-dependent enzymes predominantly localized in the cytosol, with CBS being highly expressed in the central nervous system and CSE more abundant in the cardiovascular system. In contrast, 3-MST operates primarily in the mitochondria and is involved in a distinct, transamination-based pathway. These enzymes catalyze the conversion of sulfur-containing amino acids such as cysteine and homocysteine into H_2_S, tightly regulating its production in a tissue-specific and context-dependent manner. Beyond these canonical enzymatic pathways, emerging evidence points to alternative sources of H_2_S, including lesser-known enzymes like cysteinyl-tRNA synthetase and selenium-binding protein [1], as well as nonenzymatic mechanisms that involve the reduction of elemental sulfur or thiosulfate under certain physiological conditions [1,43,44]. Additionally, the gut microbiota—particularly sulfate-reducing bacteria such as Desulfovibrio species—contribute significantly to systemic H_2_S levels, especially in the gastrointestinal tract, where microbial metabolism plays a critical role in host-microbe interactions. Once synthesized, H_2_S exhibits remarkable diffusibility, allowing it to traverse cellular membranes and act in both autocrine and paracrine fashions [1,42,43,44]. Its signaling capabilities are mediated through a variety of mechanisms, including persulfidation (S-sulfhydration) of protein cysteine residues, modulation of ion channels (e.g., ATP-sensitive potassium channels), and interaction with reactive oxygen and nitrogen species. The dynamic equilibrium between reduced and oxidized sulfur species is increasingly recognized as a critical determinant of cellular redox homeostasis and metabolic regulation. Hydrogen sulfide (H_2_S), a reduced sulfur compound, functions as a gasotransmitter with roles in vasodilation, neuromodulation, and anti-inflammatory signaling. In contrast, oxidized sulfur species such as 3′-phosphoadenosine-5′-phosphosulfate (PAPS) act as activated sulfate donors in sulfation reactions essential for detoxification and molecular signaling. While the physiological roles of H_2_S have been extensively characterized, the contribution of oxidized sulfur species like PAPS to redox balance remains underexplored. Recent studies have highlighted the importance of sulfur-based post-translational modifications, including S-sulfhydration and sulfenylation, in modulating protein function and signaling pathways, suggesting that reactive sulfur species (RSS) are not merely passive redox markers but active regulators of cellular processes [45,46]. Emerging evidence also points to the contextual functionality of sulfur compounds, where their effects depend on concentration, tissue type, and environmental stressors. For instance, a 2025 study demonstrated that H_2_S supplementation via GYY4137 restored redox balance and suppressed SARS-CoV-2 replication by activating the Nrf2/Keap1 pathway and enhancing mitochondrial bioenergetics [47]. In plants, sulfur assimilation enzymes such as adenosine-5′-phosphosulfate (APS) reductase and APS kinase have been shown to be tightly regulated under temperature stress, linking sulfur metabolism to adaptive responses [48]. Furthermore, microbial ecosystems exposed to fluctuating redox environments maintain gene expression for both oxidative and reductive sulfur pathways, indicating a flexible and resilient sulfur redox network [49]. Despite these advances, there is no universally defined optimal ratio of oxidized to reduced sulfur species. Instead, recent research suggests that sulfur redox balance is highly tissue-specific and condition-dependent. Disruption of this balance has been implicated in a variety of pathologies, including cardiovascular diseases, neurodegeneration, and cancer. Therapeutic strategies targeting sulfur metabolism—such as slow-release H_2_S donors and microbiota engineering—are being explored to restore redox homeostasis. Collectively, these findings reinforce the notion that maintaining a finely tuned sulfur redox environment—rather than achieving a fixed ratio—is essential for cellular health and stress adaptation [47,50]. These molecular interactions enable H_2_S to influence a wide spectrum of physiological processes. Functionally, H_2_S is now known to be a pleiotropic regulator involved in the maintenance of cardiovascular homeostasis (e.g., vasodilation, angiogenesis, and blood pressure regulation), neurotransmission and neuroprotection, glucose and lipid metabolism, mitochondrial bioenergetics, and immune modulation. For instance, in the cardiovascular system, H_2_S promotes endothelial cell function and inhibits vascular inflammation, while in the nervous system, it enhances synaptic plasticity and protects neurons from oxidative stress [1,44]. However, the biological actions of H_2_S are dose-dependent and context-sensitive, and dysregulation of its production or signaling has been implicated in a wide array of pathological conditions [1,44]. These include atherosclerosis, hypertension, ischemia–reperfusion injury, diabetes, inflammatory diseases, and neurodegenerative disorders such as Alzheimer’s and Parkinson’s disease. This duality—where H_2_S can be both protective and deleterious—underscores the complexity of its role in human health and disease. As a result, H_2_S has emerged as a promising therapeutic target, with ongoing research exploring the development of H_2_S-releasing drugs, enzyme modulators, and dietary interventions aimed at restoring physiological H_2_S levels. Figure 1 summarizes the various roles of H_2_S in mammalian cell pathophysiology. The field continues to evolve rapidly, offering exciting opportunities for translational applications in medicine, pharmacology, and biotechnology.

## 4. Mechanisms of Action

The therapeutic benefits of sulfur-rich mineral waters can largely be attributed to their multifaceted biochemical mechanisms, which work in concert to promote overall health and well-being. Sulfur compounds found within these unique waters have been reported to exhibit remarkable antimicrobial properties, effectively inhibiting the growth of pathogenic bacteria that contribute to various diseases and infections as well as promoting neutrophil survival and activity [51]. This powerful action can help alleviate symptoms of numerous ailments, promoting a sense of rejuvenation and vitality. Additionally, the soothing warmth of the mineral waters, combined with their rich sulfur content, can enhance circulation and provide relief to aching muscles and joints, making them a popular choice for those seeking natural therapeutic solutions [52]. For instance, sulfur-containing compounds, which are naturally occurring substances also found in various foods and plants, have been shown to play a significant role in acting against biofilm formation in oral pathogens. This intriguing property highlights their potential as valuable adjunctive treatments in dental care, especially in the ongoing battle against plaque buildup and related oral diseases. By incorporating these compounds into dental hygiene practices, we could potentially enhance the effectiveness of traditional treatments and improve overall oral health outcomes for patients [1,4,8,53]. Additionally, the presence of sulfur in various mineral waters can significantly enhance the body’s natural detoxification pathways, working diligently to support overall health and well-being. This enhancement occurs through the intricate modulation of phase II enzymes, which play a crucial role in the conjugation and elimination of harmful xenobiotics—substances that can be detrimental to our health. By promoting these vital processes, sulfur-rich mineral waters may help the body efficiently purge toxic materials, leading to improved metabolic functions [1,2,3,54]. Furthermore, the naturally occurring sulfur compounds found in spring mineral waters have been shown to significantly contribute to improved skin health and vitality. When these beneficial compounds are absorbed through the skin during a relaxing soak in the mineral-rich water, they can help alleviate various dermatological ailments, including conditions such as eczema and psoriasis, while also supporting the healing of wounds and effectively reducing skin irritation [55]. These remarkable effects stem from the powerful anti-inflammatory and desensitizing properties inherent in sulfur, which work to soothe inflamed skin, promote cellular regeneration, and enhance overall skin texture, leaving one feeling rejuvenated and revitalized [2,8,13,14,15]. Extensive research conducted by scientists and clinical dermatologists has indicated, through various studies and clinical trials, that sulfurs found in mineral waters possesses remarkable therapeutic properties that can significantly promote wound healing. Additionally, it has been shown to effectively reduce both the duration and intensity of flare-ups associated with chronic skin conditions, such as eczema and psoriasis. These findings illustrate sulfur’s potential in dermatological applications, making it a promising ingredient in skincare treatments aimed at alleviating various skin ailments and enhancing overall skin health [1,2,3,4,5,13,14,15].

The therapeutic benefits of sulfur-rich mineral waters are increasingly recognized in both traditional and modern medical practices, owing to their multifaceted biochemical and physiological mechanisms that collectively promote health and well-being. These waters, often sourced from geothermal springs, contain various forms of sulfur, including hydrogen sulfide (H_2_S), thiosulfates, and sulfates, which contribute to their unique therapeutic profile [1].

### 4.1. Antimicrobial and Immunomodulatory Effects

Sulfur compounds in mineral waters have demonstrated potent antimicrobial properties, effectively inhibiting the growth of pathogenic microorganisms such as Staphylococcus aureus, Candida albicans, and Cutibacterium acnes [2]. These effects are particularly valuable in dermatological applications, where microbial imbalance contributes to conditions like acne, eczema, and psoriasis. Moreover, sulfurous waters have been shown to enhance neutrophil survival and activity, supporting the innate immune response and contributing to the resolution of inflammation [3,12]. Sulfur-containing compounds in mineral waters exhibit significant antimicrobial activity, attributed primarily to their ability to disrupt microbial cell membranes and interfere with enzymatic functions. These compounds, including thiosulfinates, thioethers, and sulfonamides, have demonstrated efficacy against a range of pathogenic microorganisms such as Staphylococcus aureus, Candida albicans, and Cutibacterium acnes. For instance, organosulfur compounds like allicin, diallyl disulfide (DADS), and diallyl trisulfide (DATS), commonly found in sulfur-rich extracts from Allium species, have shown potent bactericidal and fungicidal properties through mechanisms involving thiol group inactivation and disruption of quorum sensing pathways [56]. These antimicrobial effects are particularly relevant in dermatological contexts, where microbial dysbiosis contributes to inflammatory skin conditions such as acne vulgaris, eczema, and psoriasis. Sulfurous waters, often used in balneotherapy, have been shown to modulate the skin microbiome and reduce colonization by pathogenic bacteria, thereby alleviating symptoms associated with these disorders [57,58]. Moreover, sulfur compounds have immunomodulatory effects. Studies indicate that sulfur-rich environments can enhance neutrophil survival and function, thereby supporting the innate immune response. This is critical in the resolution of inflammation, as neutrophils play a central role in pathogen clearance and tissue repair. For example, sulfur-containing thiodioxopiperazines such as gliotoxin have been shown to modulate inflammatory pathways and inhibit pro-inflammatory cytokines like tumor necrosis factor alpha (TNF-α) by suppressing NF-κB activity [57,58]. The pharmaceutical relevance of sulfur compounds is underscored by their prevalence in FDA-approved drugs. Sulfonamides, thioethers, and thiazoles are common motifs in antibiotics, antifungals, and anti-inflammatory agents. Their structural diversity allows for targeted activity against microbial enzymes and cell wall synthesis, making them indispensable in both topical and systemic therapies.

### 4.2. Musculoskeletal and Circulatory Benefits

The thermal properties of sulfur-rich waters—typically ranging from 30 °C to 37 °C—combined with their mineral content, promote vasodilation, improve circulation, and provide analgesic effects for individuals suffering from musculoskeletal disorders such as arthritis, fibromyalgia, and myalgia [1,42]. The warmth of the water relaxes muscles and joints, while sulfur compounds may reduce oxidative stress and inflammation in affected tissues. Sulfur-rich thermal waters, typically emerging at temperatures between 30 °C and 37 °C, possess a unique combination of physicochemical properties that contribute to their therapeutic efficacy in musculoskeletal disorders. The temperature of these waters induces vasodilation, which enhances peripheral blood flow and tissue oxygenation. This physiological response not only facilitates the removal of metabolic waste products but also promotes nutrient delivery to affected tissues, thereby supporting cellular repair and regeneration [59]. In addition to thermal effects, the mineral composition—particularly the presence of hydrogen sulfide (H_2_S)—plays a crucial role in modulating inflammatory and oxidative pathways. H_2_S is a biologically active gasotransmitter known to exert anti-inflammatory, antioxidant, and analgesic effects. It modulates the activity of NF-κB, reduces the expression of pro-inflammatory cytokines such as TNF-α and interleukin-6 (IL-6), and enhances the production of endogenous antioxidants like glutathione [59,60]. Such mechanisms are particularly beneficial in the management of rheumatic and pain-related conditions such as osteoarthritis, rheumatoid arthritis, fibromyalgia, and myalgia. Clinical studies and balneotherapy protocols have demonstrated that regular immersion in sulfurous waters leads to significant improvements in pain scores, joint mobility, and quality of life in patients with chronic musculoskeletal disorders [59]. Moreover, sulfurous thermal waters contribute to muscle relaxation and joint decompression, which are essential in reducing mechanical stress and alleviating muscle spasms. The buoyancy effect in water further supports joint unloading, making it an ideal medium for gentle physical rehabilitation and exercise [59]. The therapeutic benefits of sulfurous waters are multifactorial, encompassing not only their chemical composition and temperature but also the holistic environment of health resorts, which includes medical supervision, physical therapy, and psychological support. This integrative approach enhances the overall efficacy of treatment and supports long-term management of musculoskeletal conditions [59].

### 4.3. Oral Health and Biofilm Inhibition

Interestingly, sulfur-containing compounds also exhibit anti-biofilm activity, particularly against oral pathogens. Studies have shown that sulfur-rich mineral waters can disrupt biofilm formation by bacteria such as Streptococcus mutans and Staphylococcus aureus, which are implicated in plaque buildup and periodontal disease [2,38]. This property suggests a promising role for sulfur compounds as adjunctive agents in dental hygiene, potentially enhancing the efficacy of conventional treatments like mouth rinses and scaling. Sulfur-containing compounds have garnered increasing attention for their anti-biofilm properties, particularly in the context of oral health. Biofilms—structured microbial communities embedded in a self-produced extracellular matrix—are central to the pathogenesis of oral diseases such as dental caries, periodontitis, and peri-implantitis. These biofilms are notoriously resistant to conventional antimicrobial agents and host immune responses, making them difficult to eradicate. Recent studies have demonstrated that sulfur-rich mineral waters and sulfur-based compounds can disrupt biofilm formation by key oral pathogens, including Streptococcus mutans and Staphylococcus aureus. These bacteria are implicated in plaque accumulation, gingival inflammation, and tooth decay. The mechanism of action involves interference with quorum sensing pathways, inhibition of extracellular polysaccharide synthesis, and modulation of virulence gene expression [61]. For example, cyclic dipeptides such as cyclo (leu-pro) and hydrocinnamic acid, derived from probiotic strains like Lactiplantibacillus plantarum, have shown potent biofilm-inhibitory effects against S. mutans and Candida albicans. These compounds downregulate biofilm-associated genes such as ALS3 and HWP1, leading to reduced biofilm mass and structural integrity [62]. Additionally, their ability to penetrate biofilm matrices and alter microbial adhesion makes them promising candidates for adjunctive dental therapies, especially when integrated with conventional treatments like scaling, root planing, and antiseptic mouth rinses [63]. The potential of sulfur compounds in dental hygiene is further supported by studies on herbal and spicy extracts rich in sulfur-like molecules (e.g., eugenol, macrocarpals, carnosic acid), which exhibit synergistic biofilm eradication effects when combined. These findings suggest that sulfur-based agents could be formulated into mouthwashes, toothpaste, or coatings for dental materials to enhance antimicrobial efficacy and prevent biofilm recurrence [64].

### 4.4. Detoxification and Metabolic Support

Sulfur plays a critical role in the body’s detoxification pathways, particularly through the activation of phase II enzymes such as sulfotransferases (SULTs). These enzymes catalyze the conjugation of xenobiotics—harmful foreign substances—making them more water-soluble and easier to excrete [1,2,41]. Regular exposure to sulfur-rich mineral waters may support liver function and systemic detoxification, thereby improve metabolic resilience and reduce the burden of environmental toxins. Sulfur plays a pivotal role in the body’s detoxification systems, particularly through its involvement in Phase II biotransformation pathways, which are essential for the neutralization and excretion of xenobiotics—foreign chemical substances that may be harmful to human health. Among the key enzymes in this phase are sulfotransferases (SULTs), which catalyze the transfer of a sulfonate group (SO_3_) from the universal donor PAPS to hydroxyl, amine, or thiol groups on target molecules [65,66,67].

This conjugation reaction significantly increases the water solubility of lipophilic compounds, facilitating their renal or biliary excretion. SULTs are widely expressed in the cytosol of liver cells, where they metabolize a broad spectrum of substrates, including drugs, environmental toxins, steroid hormones, neurotransmitters, and bile acids. Their activity is crucial not only for detoxification but also for regulating the biological activity of endogenous molecules, such as estrogens and catecholamines [65,66,67]. Importantly, interindividual variability in SULT expression and function—due to genetic polymorphisms or environmental influences—can affect susceptibility to toxins and drug efficacy. This underscores the importance of maintaining optimal SULT activity for metabolic resilience and toxicological defense [67]. Regular exposure to sulfur-rich mineral waters may support these detoxification processes by providing bioavailable sulfur species that serve as precursors for PAPS synthesis and other sulfur-dependent metabolic reactions. H_2_S, a key component in many sulfurous waters, has been shown to modulate redox balance, inflammatory signaling, and cellular stress responses, which are intimately linked to hepatic detoxification capacity [65,66,67].

### 4.5. Dermatological Actions

One of the most well-documented benefits of sulfur-rich mineral waters is their impact on skin health. When absorbed through the skin during balneotherapy, sulfur compounds exert anti-inflammatory, keratolytic, and antioxidant effects that help alleviate chronic skin conditions such as eczema, psoriasis, seborrheic dermatitis, and acne. Clinical studies, including a pilot trial at Lake Hévíz in Hungary, have shown that sulfur thermal water can improve Psoriasis Area and Severity Index (PASI) scores and modulate the skin microbiome, suggesting both symptomatic and microbiological benefits [4,5,14,15]. Sulfur-rich mineral waters have long been recognized for their therapeutic potential in dermatology, particularly when administered through balneotherapy. The absorption of sulfur compounds through the skin during immersion in thermal waters facilitates a multifaceted biological response, including anti-inflammatory, keratolytic, and antioxidant effects. These mechanisms are especially beneficial in the management of chronic inflammatory skin conditions such as eczema, psoriasis, seborrheic dermatitis, and acne [13,32]. The anti-inflammatory action of sulfur compounds is mediated by the modulation of cytokine production and inhibition of NF-κB signaling, which reduces the infiltration of immune cells and the release of pro-inflammatory mediators. The keratolytic effect, attributed to H_2_S and other sulfur derivatives, promotes desquamation and normalization of epidermal turnover, which is particularly useful in hyperkeratotic conditions like psoriasis and seborrheic dermatitis. Additionally, sulfur compounds act as antioxidants, scavenging reactive oxygen species (ROS) and enhancing the activity of endogenous antioxidant enzymes such as glutathione peroxidase, thereby protecting skin cells from oxidative damage [32,68,69]. Clinical evidence supports these therapeutic claims. A pilot study conducted at Lake Hévíz in Hungary, a natural sulfur thermal lake, demonstrated significant improvements in Psoriasis Area and Severity Index (PASI) scores following a 3-week balneotherapy regimen. Participants underwent 30 min sessions five times per week, and results showed not only symptomatic relief but also modulation of the skin microbiome, with shifts in bacterial genera such as Leptolyngbya and Flavobacterium. These microbiological changes suggest that sulfurous waters may help restore microbial balance, which is increasingly recognized as a key factor in skin health and disease [70]. Further support comes from the PSOTHERMES randomized clinical trial, which evaluated spa therapy in five French resorts. The study found that patients with plaque psoriasis who received immediate spa therapy showed significant improvements in quality of life, pruritus, and PASI scores compared to controls. These benefits persisted at 12-month follow-up, underscoring the long-term efficacy of sulfur-based balneotherapy [71]. Taken together, the therapeutic effects of sulfur-rich mineral waters are multidimensional, encompassing antimicrobial defense, immune modulation, detoxification, circulatory enhancement, and dermatological healing. These waters offer a natural, non-invasive adjunct to conventional therapies, with growing evidence supporting their use in clinical balneotherapy, dermocosmetics, and preventive medicine.

## 5. Therapeutic Applications

Sulfur-enriched mineral waters are supported by clinical and mechanistic evidence as complementary therapies for rheumatic and musculoskeletal disorders, including osteoarthritis, rheumatoid arthritis, and fibromyalgia. Reported benefits consistently include reductions in pain, inflammation, and stiffness, alongside gains in mobility and quality of life, reflecting the combined influence of thermal exposure and immersion mechanics, as well as the biochemical activity of dissolved sulfur species (H_2_S/HS^−^/S^2−^) on inflammatory and redox pathways [59,72,73,74,75]. This therapeutic effect has been linked to the dual action of both the thermal and sulfur components present in the mineral waters, where the warm thermal water applications enhance blood flow, stimulate circulation, and effectively reduce muscle tension [4,5]. Meanwhile, the sulfur itself actively modulates inflammatory mediators, contributing to a decrease in the severity of symptoms associated with chronic conditions [2,4,52,53,76,77]. Together, these elements create a holistic approach to pain management and rehabilitation, offering hope to those seeking relief from the relentless grip of rheumatic ailments. In more specialized scenarios, particularly within the realm of medical rehabilitation, mineral waters that are rich in sulfur compounds have been effectively utilized as a therapeutic tool to significantly boost recovery processes in post-operative patients who are seeking to regain their health and functionality. The potential absorption of sulfur through the skin [55,78,79,80,81,82,83] during these mineral baths may facilitate the promotion of systemic circulation, which in turn leads to an enhanced supply of essential nutrients to the healing areas of the body, ultimately promoting a more efficient and accelerated recovery [1,8].

Mechanistically, H_2_S has been described to modulate cytokine profiles (e.g., promoting anti-inflammatory signaling), engage antioxidant defenses (e.g., Keap1–Nrf2 axis), and support microvascular function and tissue repair—mechanisms that plausibly contribute to symptom relief after spa cycles. Health-resort medicine literature also discusses percutaneous uptake of sulfur species during balneotherapy, which may add local and limited systemic effects, depending on water chemistry and exposure parameters [59,75].

Clinically, controlled studies in osteoarthritis suggest that courses of balneotherapy with mineral waters can improve pain and function beyond standard care or tap-water comparators, with some durability beyond the treatment window. Broader reviews in rheumatology concur that hydro/balneotherapy alleviates pain, improves joint mobility, and enhances overall well-being when integrated as an adjunct to conventional management [72,73,74].

In specialized rehabilitation settings, adjunctive electrotherapies using sulfurous waters have shown promise. A prospective clinical study in knee osteoarthritis comparing iontophoresis with Ilidža (Sarajevo) thermomineral sulfur water versus classic galvanization reported larger reductions in pain and better gains in mobility in the sulfur-water arm, supporting the practical value of transdermal sulfur delivery in routine rehabilitation programs [72].

Finally, comprehensive overviews emphasize that benefits extend beyond physicochemical factors: structured spa environments and warm-water immersion can enhance psychological relaxation and stress resilience, contributing to overall quality of life improvements. Accordingly, sulfur-enriched mineral water therapy can be reasonably integrated into multidisciplinary care plans for degenerative and inflammatory musculoskeletal conditions as a safe, supportive adjunct when appropriately indicated [59,73,74,75].

## 6. Health Implications

Given that sulfur compounds play crucial and multifaceted roles in maintaining essential cellular functions and supporting various biochemical pathways within the body, their remarkable therapeutic properties found in mineral waters underscore their significant importance in the realm of public health. Enhanced access to sulfur-rich mineral water sources has the potential to deliver a myriad of beneficial health impacts to communities, particularly in regions that are predisposed to chronic inflammatory diseases, such as arthritis, respiratory and cardiometabolic conditions and even neurologic and cognitive conditions linked to aging [83,84,85,86,87,88,89,90,91,92,93,94,95]. Furthermore, comprehensive educational outreach initiatives that focus on the many advantages of sulfur mineral baths could serve to bolster preventive health strategies, while also encouraging lifestyle modifications that aim to reduce inflammation and combat oxidative stress, which is often linked to a range of health issues. Moreover, regulatory bodies could greatly benefit from considering these sulfide-rich waters in therapeutic contexts, especially when developing guidelines that pertain to their safe and effective use in both clinical and wellness settings. This is particularly relevant in an era where holistic and natural treatments are increasingly sought after. Ongoing and rigorous research into the specific health impacts, as well as the optimal dosages of sulfur baths and mineral waters, can further refine public health recommendations, ensuring that individuals can make informed choices about their health and well-being while harnessing the remarkable benefits that these naturally occurring compounds offer. Ultimately, a stronger understanding of sulfur’s role and its applications in therapeutic practices could pave the way for innovative public health initiatives aimed at improving community health outcomes across diverse populations.

## 7. Future Directions

The ongoing, multidisciplinary research into sulfur compounds present in various mineral waters represents a promising frontier in both health sciences and environmental ecology. These naturally occurring compounds, particularly hydrogen sulfide, have garnered increasing attention due to their potential therapeutic effects, especially in the context of chronic diseases that are closely associated with oxidative stress and systemic inflammation. Given the rising global burden of conditions such as cardiovascular disease, autoimmune disorders, and metabolic syndromes, the biological activity of sulfur compounds warrants rigorous and targeted investigation. Preliminary studies suggest that hydrogen sulfide exhibits generally inhibitory effects on platelet function and others in vivo processes [96,97], indicating a possible role in modulating vascular health and thrombosis risk. However, the underlying mechanisms remain insufficiently understood. Future research should aim to elucidate how sulfur compounds interact with human cells, particularly in regulating inflammatory pathways, oxidative stress responses, and immune system modulation. Key areas of focus include the absorption kinetics of these compounds—whether through dermal exposure or gastrointestinal uptake—and their subsequent metabolic transformations within the body. To fully harness the therapeutic potential of sulfur-rich mineral waters, it is essential to identify the optimal concentrations, exposure durations, and water compositions that yield the most beneficial outcomes. Comparative studies are needed to determine which types of sulfur waters offer superior efficacy for specific health conditions. High-quality randomized controlled trials will be critical in validating their use for ailments such as arthritis, psoriasis, eczema, and respiratory disorders. Moreover, long-term safety assessments are necessary to evaluate potential side effects, contraindications, and cumulative impacts of regular exposure. Innovative research methodologies [98], including advanced analytical techniques such as metabolomics, microbiome profiling, and high-resolution spectroscopy, are strongly encouraged. These multidisciplinary approaches offer valuable insights into the intricate interactions between sulfur compounds and diverse microbial communities, illuminating their influence on both localized physiological processes and broader systemic health outcomes. Understanding these dynamics is crucial for advancing therapeutic strategies and public health interventions. Simultaneously, public health initiatives should prioritize raising awareness about the potential benefits of natural mineral waters, particularly those rich in sulfur. By fostering community engagement and encouraging responsible consumption, such efforts can contribute to healthier lifestyle choices and improved population well-being. Educational campaigns, wellness programs, and collaborations with healthcare providers can empower individuals to make informed decisions, bridging the gap between scientific knowledge and everyday practice. Integrating sulfur-based therapies into conventional medical frameworks is a key step toward holistic healthcare. Researchers and clinicians could explore adjunctive and synergistic treatment models that combine sulfur-rich mineral water therapies with established interventions such as antibiotics, anti-inflammatory agents, or dermatological treatments. These integrative approaches may enhance therapeutic efficacy, reduce side effects, and support long-term health outcomes. Similarly, pairing these waters with other natural modalities—such as mud therapy or herbal medicine—could yield synergistic effects that amplify health benefits. Ultimately, a holistic and evidence-based approach to sulfur compound research will pave the way for novel interventions in areas of unmet medical need, contributing to a more integrative and sustainable healthcare paradigm [35,98,99].

## 8. Conclusions

The evidence supporting the health-promoting properties of natural sulfur compounds found in mineral waters is both compelling and increasingly substantiated by a growing body of interdisciplinary research. These compounds, long revered in traditional medicine and now validated through modern scientific inquiry, exhibit a multifaceted therapeutic profile that includes anti-inflammatory, antioxidant, antimicrobial, and immunomodulatory actions. Such properties position sulfur-rich mineral waters as promising adjuncts in the management of a wide spectrum of health conditions, ranging from chronic inflammatory diseases like arthritis and psoriasis to respiratory ailments, metabolic dysfunctions, and microbial infections. One of the most striking aspects of sulfur compounds is their ability to modulate biological pathways at both the cellular and systemic levels. By attenuating oxidative stress and inflammatory cascades, enhancing detoxification via phase II enzymes, and supporting immune cell function, these compounds contribute to the restoration of physiological balance and resilience. Moreover, their topical and systemic effects—particularly in dermatological and musculoskeletal applications—underscore their versatility and potential for integration into both preventive and therapeutic health strategies. The incorporation of sulfur-rich mineral water therapies into mainstream wellness and medical practices offers a holistic, non-invasive, and nature-based approach to health promotion. This is especially relevant in an era where integrative medicine and sustainable healthcare solutions are gaining traction. Balneotherapy, spa medicine, and mineral water-based treatments could serve as valuable complements to pharmacological interventions, particularly for individuals seeking alternatives with fewer side effects or those managing chronic conditions. However, despite the promising clinical and anecdotal outcomes, the scientific validation and optimization of these therapies require further attention. Rigorous, large-scale, and well-controlled studies are essential to elucidate the precise mechanisms of action, determine optimal dosing and exposure parameters, and assess long-term safety and efficacy across diverse populations. Additionally, fostering interdisciplinary collaboration among clinicians, biochemists, public health experts, and environmental scientists will be essential for advancing this emerging field. Such partnerships can drive innovation, ensure scientific rigor, and facilitate the translation of research findings into practical health solutions. In conclusion, sulfur-rich mineral waters represent a promising natural reservoir of therapeutic potential. With sustained scientific inquiry and thoughtful integration into healthcare systems, these resources may offer meaningful benefits for both individual well-being and broader public health outcomes. Realizing their full potential will require not only robust research and clinical validation but also a renewed appreciation for the healing capacities found in nature. By bridging traditional knowledge with modern science, we can unlock new pathways toward holistic and sustainable health practices.

## Data Availability

No new data were created or analyzed in this study. Data sharing is not applicable to this article.

## References

[1] Cirino G., Szabo C., Papapetropoulos A. (2023). Physiological roles of Hydrogen Sulfide in mammalian cells, tissues and organs. Physiol. Rev..

[2] Szabo C. (2007). Hydrogen Sulfide and its therapeutic potential. Nat. Rev. Drug Discov..

[3] Nahzli D., Papapetropoulos A., Toliver-Kinsky T., Szabo C. (2020). Hydrogen sulfide: An endogenous regulator of the immune system. Pharm. Res..

[4] Karagulle M.Z., Karagulle M. (2020). Effects of drinking natural hydrogen sulfide (H2S) waters: A systematic review of in vivo animal studies. Int. J. Biometeorol..

[5] Cheleschi S., Gallo I., Tenti S. (2020). A comprehensive analysis to understand the mechanism of action of balneotherapy: Why, how, and where they can be used? Evidence from in vitro studies performed on human and animal samples. Int. J. Biometeorol..

[6] Vaccarezza M., Vitale M. (2010). Crenotherapy: A neglected resource for human health now re-emerging on sound scientific concepts. Int. J. Biometeorol..

[7] Scapagnini G., Davinelli S., Fortunati N.A., Zella D., Vitale M., Rattan S.I.S., Le Bourg E. (2014). Thermal Hydrotherapy as Adaptive Stress Response: Hormetic Significance, Mechanisms, and Therapeutic Implications. Hormesis in Health and Disease.

[8] Szabo C. (2022). Novel Regulatory Roles of Hydrogen Sulfide in Health and Disease. Biomolecules.

[9] Sp N., Kang D., Kim H., Rugamba A., Jo E., Park J., Jang K. (2021). Natural sulfurs inhibit LPS-induced inflammatory responses through NF-κb signaling in ccd-986sk skin fibroblasts. Life.

[10] Mirandola P., Gobbi G., Micheloni C., Vaccarezza M., Di Marcantonio D., Ruscitti F., de Panfilis G., Vitale M. (2011). Hydrogen sulfide inhibits IL-8 expression in human keratinocytes via MAP kinase signaling. Lab. Investig..

[11] Burguera E., Vela-Anero Á., Gato-Calvo L., Vaamonde-García C., Meijide-Faílde R., Blanco F. (2019). Hydrogen sulfide biosynthesis is impaired in the osteoarthritic joint. Int. J. Biometeorol..

[12] Maccarone M.C., Magro G., Solimene U., Scanu A., Masiero S. (2021). From in vitro research to real life studies: An extensive narrative review of the effects of balneotherapy on human immune response. Sport. Sci. Health.

[13] Carubbi C., Gobbi G., Bucci G., Gesi M., Vitale M., Mirandola P. (2013). Skin, Inflammation and sulfurous waters: What is known, what is believed. Eur. J. Inflamm..

[14] Gross-Amat O., Guillen M., Gimeno J.P., Salzet M., Lebonvallet N., Misery L., Auxenfans C., Nataf S. (2020). Molecular Mapping of Hydrogen Sulfide Targets in Normal Human Keratinocytes. Int. J. Mol. Sci..

[15] Karagulle M.Z., Karagulle M., Kilic S., Sevinc H., Dundar C., Turkoglu M. (2018). In vitro evaluation of natural thermal mineral waters in human keratinocyte cells: A preliminary study. Int. J. Biometeorol..

[16] Pozsgai G., Benko R., Bartho L., Horvath K., Pinter E. (2015). Thermal Spring water drinking attenuates dextran-sulfate-sodium-induced colitis in mice. Inflammopharmacology.

[17] Prandelli C., Parola C., Buizza L., Delbarba A., Marziano M., Salvi V., Zacchi V., Memo M., Sozzani S., Calza S. (2013). Sulfurous thermal water increases the release of the anti-inflammatory cytokine IL-10 and modulates antioxidant enzyme activity. Int. J. Immunopath Pharm..

[18] Sieghart D., Listz M., Wanivenhaus A., Broll H., Kiener H., Klosch B., Steiner G. (2015). Hydrogen sulphide decreases Il-beta-induced activation of fibroblast-like synoviocytes from patients with osteoarthritis. J. Cell Mol. Med..

[19] Lopalco M., Proia A.R., Fraioli A., Serio A., Cammarella I., Petraccia L., Grassi M. (2004). Therapeutic effect of the association between pulmonary ventilation and aerosol-inhalation with sulphureous mineral water in the chronic bronchopneumopathies. Clin. Ther..

[20] Kovacs C., Pecze M., Tihanyi A., Kovacs L., Balogh S., Bender T. (2012). The effect of sulphurous water in patients with osteoarthritis of hand. Double-blind, randomized, controlled follow-up study. Clin. Rheumatol..

[21] Codish S., Dobrovinsky S., Abu Shakra M., Flusser D., Sukenik S. (2005). Spa therapy for ankylosing spondylltis at the Dead Sea. Isr. Med. Assoc. J..

[22] Goszcz A., Kostka-Trabka E., Grodzinska L., Stawinski M., Bieron K., Jachym R., Kucharski K., Gryglewski R.J. (1997). The effect of treatment with sulphur water from the spring in Wiesław in Busko-Solec on levels of lipids, the fibrinolytic system and thrombogenic platelet function in patients with arteriosclerosis. Pol. Merkur. Lek..

[23] Soria M., Gonzales-Haro C., Esteva S., Escanero J.F., Pin J.R. (2014). Effect of sulphurous mineral water in haematological and biochemical markers of muscle damage after an endurance exercise in well-trained athletes. J. Sports Sci..

[24] Kovacs C., Boszik A., Pecze M., Borbely I., Fogarasi A., Kovacs L., Tefner I.K., Bender T. (2016). Effects of sulfur bath on hip osteoarthritis: A randomized, controlled, single-blind, follow-up trial: A pilot study. Int. J. Biometeorol..

[25] Salami A., Dellepiane M., Crippa B., Mora F., Guastini L., Jankowska B., Mora R. (2008). Sulphurous water inhalations in the prophylaxis of recurrent upper respiratory tract infections. Int. J. Pediatr. Otorhinolaryngol..

[26] Mancini S., Piccinetti A., Nappi G., Mancini S., Caniato A., Coccheri S. (2003). Clinical, functional and quality of life changes after balneokinesis with sulphurous water in patients with varicose veins. VASA.

[27] Branco M., Rego N.N., Silva P.H., Archanjo I.E., Ribeiro M.C., Trevisani V.F. (2016). Bath thermal waters in the treatment of knee osteoarthritis: A randomized controlled clinical trial. Eur. J. Phys. Rehabil. Med..

[28] Staffieri A., Marino F., Staffieri C., Giacomelli L., D’Alessandro E., Maria Ferraro S., Fedrazzoni U., Marioni G. (2008). The effects of sulfurous-arsenical-ferruginous thermal water nasal irrigation in wound healing after functional endoscopic sinus surgery for chronic rhinosinusitis: A prospective randomized study. Am. J. Otolaryngol..

[29] Constantino M., Filippelli A., Quenau P., Nicolas J.P., Coiro V. (2012). Sulphur mineral water and SPA therapy in osteoarthritis. Therapies.

[30] Ottaviano G., Marioni G., Giacomelli L., La Torre F.B., Staffieri C., Marchese-Ragona R., Staffieri A. (2012). Smoking and chronic rhinitis: Effects of nasal irrigations with sulfurous-arsenical-ferruginous thermal water: A prospective, randomized, double-blind study. Am. J. Otolaryngol..

[31] Benedetti S., Benvenuti F., Nappi G., Fortunati N.A., Marino L., Aureli T., De Luca S., Pagliarani S., Canestrari F. (2009). Antioxidative effects of sulfurous mineral water: Protection against lipid and protein oxidation. Eur. J. Clin. Nutr..

[32] Kanwal S., Osman E.Y., Khiari I. (2025). Comprehensive review of dermatological and cosmeceutical manifestations of thermal water and future insights. Int. J. Biometeorol..

[33] Verhagen A.P., Bierma-Zeinstra S.M., Boers M., Cardoso J.R., Lambeck J., de Bie R., de Vet H.C. (2015). Balneotherapy (or spa therapy) for rheumatoid arthritis. Cochrane Database Syst. Rev..

[34] Ottaviano G., Marioni G., Staffieri C., Giacomelli L., Marchese-Ragona R., Bertolin A., Staffieri A. (2011). Effects of sulfurous, salty, bromic, iodic thermal water nasal irrigations in nonallergic chronic rhinosinusitis: A prospective, randomized, double-blind, clinical, and cytological study. Am. J. Otolaryngol..

[35] Crucianelli S., Mariano A., Valeriani F., Cocomello N., Gianfranceschi G., Baseggio Conrado A., Moretti F., Scotto d’Abusco A., Mennuni G., Fraioli A. (2024). Effects of sulphur thermal water inhalations in long-COVID syndrome: Spa-centred, double-blinded, randomised case-control pilot study. Clin. Med..

[36] Contoli M., Gnesini G., Forini G., Marku B., Pauletti A., Padovani A., Casolari P., Taurino L., Ferraro A., Chicca M. (2013). Reducing agents decrease the oxidative burst and improve clinical outcomes in COPD patients: A randomised controlled trial on the effects of sulphurous thermal water inhalation. Sci. World J..

[37] Szabo C. (2018). A timeline of hydrogen sulfide (H2S) research: From environmental toxin to biological mediator. Biochem. Pharmacol..

[38] Warenycia M.W., Goodwin L.R., Benishin C.G., Reiffenstein R.J., Grancom D.M., Taylor J.D., Dieken F.P. (1989). Acute hydrogen sulfide poisoning. Demonstration of selective uptake of sulfide by the brainstem by measurement of brain sulfide levels. Biochem. Pharmacol..

[39] Rana A., Katiyar A., Arun A., Berrios J.N., Kumar G. (2025). Natural sulfur compounds in mental health and neurological disorders: Insights from observational and intervention studies. Front. Nutr..

[40] Mustafa A.K., Gadalla M.M., Sen N., Kim S., Mu W., Gazi S.K., Barrow R.K., Yang G., Wang R., Snyder S.H. (2009). H2S Signals Through Protein S-Sulfhydration. Sci. Signal..

[41] Kimura H. (2021). Hydrogen Sulfide (H_2_S) and Polysulfide (H_2_Sn) Signaling: The First 25 Years. Biomolecules.

[42] Munteanu C., Turnea M.A., Rotariu M. (2023). Hydrogen Sulfide: An Emerging Regulator of Oxidative Stress and Cellular Homeostasis. Antioxidants.

[43] Zhu Y.Z., Wang R. (2018). Production of H_2_S—The l-cysteine/CSE-CBS-MST/H_2_S System. Gasotransmitters.

[44] Rose P., Moore P.K., Zhu Y.Z. (2017). H2S biosynthesis and catabolism: New insights from molecular studies. Cell Mol. Life Sci..

[45] Menendez C. (2025). Revisiting Redox Biology: The Role of Sulfur-Based Modifications in Cellular Metabolism. Biochem. Anal. Biochem..

[46] Hou Y., Lv B., Du J., Jin H., Yi Y., Huang Y. (2025). Sulfide regulation and catabolism in health and disease. Signal Transduct. Target. Ther..

[47] Andrés C.M.C., Lobo F., Pérez de la Lastra J.M., Munguira E.B., Juan C.A., Pérez Lebeña E. (2025). Reactive Sulfur Species and Protein Persulfidation: An Emerging Redox Axis in Human Health and Disease. Curr. Issues Mol. Biol..

[48] Kopriva S., Rahimzadeh Karvansara P., Takahashi H. (2024). Adaptive modifications in plant sulfur metabolism over evolutionary time. J. Exp. Bot..

[49] Luo W., Zhao M., Dwidar M., Gao Y., Xiang L., Wu X., Medema M.H., Su X., Li X., Schafer H. (2024). Microbial assimilatory sulfate reduction-mediated H_2_S: An overlooked role in Crohn’s disease development. Microbiome.

[50] Sies H., Mailloux R.J., Jakob U. (2024). Fundamentals of redox regulation in biology. Nat. Rev. Mol. Cell Biol..

[51] Rinaldi L., Gobbi G., Pambianco M., Micheloni C., Mirandola P., Vitale M. (2006). Hydrogen sulfide prevents apoptosis of human PMN via inhibition of p38 and caspase 3. Lab. Investig..

[52] Lopes S., Morgado S., Gomes A., Lopes P., Couto P., Correia M., Foler-Fraile J., Veiga N. (2024). Unraveling the benefits of thermal waters enhancing oral health: A pilot study. BMC Oral. Health.

[53] Altaany Z., Alkaraki A., Abu-Siniyeh A., Momani W., Taani O. (2019). Evaluation of antioxidant status and oxidative stress markers in thermal sulfurous springs residents. Heliyon.

[54] Carubbi C., Masselli E., Calabrò E., Bonati E., Galeone C., Andreoli R., Goldoni M., Corradi M., Sverzellati N., Pozzi G. (2019). Sulfurous thermal water inhalation impacts respiratory metabolic parameters in heavy smokers. Int. J. Biometeorol..

[55] Cacciapuoti S., Luciano M.A., Megna M., Annunziata M.C., Napolitano M., Patruno C., Scala E., Colicchio R., Pagliuca C., Salvatore P. (2020). The Role of Thermal Water in Chronic Skin Diseases Management: A Review of the Literature. J. Clin. Med..

[56] Feknous N., Boumendjel M., Leblab F.Z. (2024). Updated Insights on the Antimicrobial Activities of Allium Genus. Russ. J. Bioorg Chem..

[57] Hai Y., Wei M.-Y., Wang C.-Y., Gu Y.-C., Shao C.-L. (2021). The intriguing chemistry and biology of sulfur-containing natural products from marine microorganisms. Mar. Life Sci. Technol..

[58] Scott K.A., Njardarson J.T. (2018). Analysis of US FDA-Approved Drugs Containing Sulfur Atoms. Top. Curr. Chem..

[59] Amado F.M.L., Silva E.A.F., Gomes C.S., Routureau M. (2021). Healing Sulfurous Thermal Waters in Health Resort Medicine: Therapies, Indications, and Contraindications. Minerals Latu Sensu and Human Health.

[60] Bhatia M. (2015). H_2_S and inflammation: An overview. Handb. Exp. Pharmacol..

[61] Jacob M.M., Dhanya K.C., Lhiri D., Nag M., Bhattacharya D., Pati S., Sarkar S. (2025). Antibiofilm Activity of Natural Compounds. Bioactive Ingredients for Healthcare Industry Advances in Therapeutic Applications Volume 2.

[62] Li J., Zhang Q., Zhao J., Zhang H., Chen W. (2022). Streptococcus mutans and Candida albicans Biofilm Inhibitors Produced by Lactiplantibacillus plantarum CCFM8724. Curr. Microbiol..

[63] Nwankwo N.E., David J.C. (2025). A review of sulfur-containing compounds of natural origin with insights into their Pharmacological and toxicological impacts. Discov. Chem..

[64] Govindarajan D.K., Mohanarangam M., Kadivelu L., Sivaramalingam S.S., Jothivel D., Ravichandran A., Rariasamy S., Kandaswamy K. (2025). Biofilms and Oral Health: Nanotechnology for Biofilm Control. Discov. Nano.

[65] Glatt H., Schwab M. (2017). Sulfotransferases. Encyclopedia of Cancer.

[66] Chen C.-H., Chen C.-H. (2024). Phase II Detoxification Enzymes. Activation and Detoxification Enzymes Functions and Implications.

[67] Grant D.M. (1999). Detoxification Pathways in the Liver. J. Inherit. Metab..

[68] Huang A., Seite S., Adar T. (2018). The use of balneotherapy in dermatology. Clin. Dermatol..

[69] Carbajo J.M., Maraver F. (2017). Sulphurous mineral waters: New applications for health. Evid.-Based Complement. Altern. Med..

[70] Kulisch A., Mando Z., Sandor E., Lengyel Z., Illes A., Kosa J., Arvai K., Lakatos P., Tobias B., Papp M. (2023). Evaluation of Lake Hévíz sulfur thermal water on skin microbiome in plaque psoriasis: An open label, pilot study. Int. J. Biometeorol..

[71] Beylot-Barry M., Mahe E., Rolland C., de la Breteque M.A., Eychenne C., Charles J., Payen C., Machet L., Vermorel C., Foote A. (2022). Evaluation of the benefit of thermal spa therapy in plaque psoriasis: The psothermes randomized clinical trial. Int. J. Biometeorol..

[72] Skopljak A. (2024). The Therapeutic Effects of Thermo-Mineral Water Ilidža-Sarajevo in Osteoarthritis of the Knee. World J. Adv. Res. Rev..

[73] Tanović E. (2019). Dilemmas in the Application of Hydrotherapy and Balneotherapy. Acta Sci. Med. Sci..

[74] Fabiani D., Partsch R., Casale R., Matucci Cerinic M. (1996). Rheumatologic Aspects of Mineral Water. Clin. Dermatol..

[75] Bekaryssova D., Yessirkepov M., Imanbaeva A.D. (2024). Water-Based Interventions in Rheumatic Diseases: Mechanisms, Benefits, and Clinical Applications. Rheumatol. Int..

[76] Bernetti A., Mangone M., Alviti F., Paolucci T., Attanasi C., Murgia M., Di Sante L., Agostini F., Vitale M., Paoloni M. (2020). Spa therapy and rehabilitation of musculoskeletal pathologies: A proposal for best practice in Italy. Int. J. Biometeorol..

[77] Ariani A., Bedogni G., Biasi G., Cozzi F., Formisano S., Gorla R., Guiducci S., Maccarone M.C., Masiero S., Montalbano S. (2025). “Thermalism, Rheumatic Disease” study group of the Italian Society of Rheumatology (SIR). Balneotherapy in Fibromyalgia Syndrome: Protocol of “FIBROTHERM”, a prospective multi-center, two-cohort observational study. Int. J. Biometeorol..

[78] Pozzi G., Gobbi G., Masselli E., Carubbi C., Presta V., Ambrosini L., Vitale M., Mirandola P. (2022). Buffering Adaptive Immunity by Hydrogen Sulfide. Cells.

[79] Viegas J., Esteves A.F., Cardoso E.M., Arosa F.A., Vitale M., Taborda-Barata L. (2019). Biological Effects of Thermal Water-Associated Hydrogen Sulfide on Human Airways and Associated Immune Cells: Implications for Respiratory Diseases. Front. Public. Health.

[80] Neesby T.E., Koff A., Pircio A.W. (1955). A preliminary note on the absorption of sulfur and the polythinates into intact skin. J. Am. Pharm. Assoc..

[81] Neesby T.E., Pircio A.W., Grattan J.F. (1957). The absorption of sulfur compounds from externally deposited polythionates by the skin of the rat. J. Am. Pharm. Assoc..

[82] Haftek M., Abdayem R., Guyonnet-Debersac P. (2022). Skin Minerals: Key Roles of Inorganic Elements in Skin Physiological Functions. Int. J. Mol. Sci..

[83] Antonelli M., Fasano F., Veronesi L., Donelli D., Vitale M., Pasquarella C. (2024). Balneotherapy and cortisol levels: An updated systematic review and meta-analysis. Int. J. Biometeorol..

[84] Braga P.C., Dal Sasso M., Culici M., Spallino A., Marabini L., Bianchi T., Nappi G. (2010). Effects of sulpurous water on human neutrophil elastase release. Ther. Adv. Resp. Dis..

[85] Calzetta L., Di Daniele N., Chetta A., Vitale M., Gholamalishahi S., Cazzola M., Rogliani P. (2024). The Impact of Thermal Water in Asthma and COPD: A Systematic Review According to the PRISMA Statement. J. Clin. Med..

[86] Jeddi S., Gheibi S., Afzali H., Carlstrom M., Kashfi K., Ghasemi A. (2022). Hydrogen sulfide potentiates the protective effects of nitrite against myocardial ischemia-reperfusion injury in type 2 diabetic rats. Nitric Oxide.

[87] Zapolsky T., Kornecki W., Jaroszynski A. (2024). The Influence of Balneotherapy Using Salty Sulfide–Hydrogen Sulfide Water on Selected Markers of the Cardiovascular System: A Prospective Study. J. Clin. Med..

[88] Hajiaqaei M., Ranjbaran M., Kadkhodaee M., Shafie A., Abdi A., Lorian K., Kianian F., Seifi B. (2024). Hydrogen sulfide upregulates hypoxia inducible factors and erythropoietin production in chronic kidney disease induced by 5/6 nephrectomized rats. Mol. Biol. Rep..

[89] Tanczos B., Vass V., Szabo E., Lovas M., Kattoub R.G., Bereczki I., Borbas A., Herczegh P., Tosaki A. (2024). Effects of H_2_S-donor ascorbic acid derivative and ischemia/reperfusion-induced injury in isolated rat hearts. Eur. J. Pharm. Sci..

[90] Li T., Liu H., Xue H., Zhang J., Han X., Yan S., Bo S., Liu S., Yuan L., Deng L. (2017). Neuroprotective Effects of Hydrogen Sulfide Against Early Brain Injury abd Secondary Cognitive Deficits Following Subarachnoid Hemorrhage. Brain Pathol..

[91] Lopez-Preza F.I., Huerta de la Cruz S., Santiago-Castaneda C., Silva-Velasco D.L., Beltran-Ornelas J.H., Tapia-Martinez J., Sanchez-Lopez A., Rocha L., Centurion. D. (2023). Hydrogen sulfide prevents the vascular dysfunction induced by severe traumatic brain injury in rats by reducing reactive oxygen species and modulating eNOS and H_2_S-synthesizing enzyme expression. Life Sci..

[92] Giuliani D., Ottani A., Zaffe D., Galantucci M., Strinati F., Lodi R., Guarini S. (2013). Hydrogen sulfide slows down progression of experimental Alzheimer’s disease by targeting multiple pathophysiological mechanisms. Neurobiol. Learn. Mem..

[93] Chen H., Sun H., Hua W., Chang H., Chen W., Ma S. (2024). Exogenous hydrogen sulfide ameliorates diabetes-associated cognitive dysfunction by regulating the nrf-2/HO-1 axis and the NLRP3 inflammasome pathway in diabetic rats. Eur. J. Pharm..

[94] Munteanu C., Iordan D.A., Hoteteu M., Popescu C., Postoiu R., Onu I., Onose G. (2023). Mechanistic Intimate Insights into the Role of Hydrogen Sulfide in Alzheimer’s Disease: A Recent Systematic Review. Int. J. Mol. Sci..

[95] Shayea A.M.F., Mousa A.M.A., Renno W.M., Shaban Nadar M., Qabazard B., Yousif M.H.M. (2020). Chronic Treatment with Hydrogen Sulfide Donor GYY4137 Mitigates Microglial and Astrocyte Activation in the Spinal Cord of Streptozotocin-Induced Diabetic Rats. J. Neuropathol. Exp. Neurol..

[96] Truss N.J., Warner T.D. (2011). Gasotransmitters and platelets. Pharmacol. Ther..

[97] Gobbi G., Mirandola P., Tazzari P.L., Ricci F., Caimi L., Cacchioli A., Papa S., Conte R., Vitale M. (2003). Flow cytometry detection of serotonin content and release in resting and activated platelets. Br. J. Haematol..

[98] Pozzi G., Masselli E., Gobbi G., Mirandola P., Taborda-Barata L., Ampollini L., Carbognani P., Micheloni C., Corzza F., Galli D. (2021). Hydrogen Sulfide Inhibits TMPRSS2 in Human Airway Epithelial Cells: Implications for SARS-CoV-2 Infection. Biomedicines.

[99] Viegas J., Cardoso E.M., Bonneau L., Esteves A.F., Ferreira C.L., Alves G., Santos-Silva A., Vitale M., Arosa F.A., Taborda-Barata L. (2024). A Novel Bionebulizer Approach to Study the Effects of Natural Mineral Water on a 3D In Vitro Nasal Model from Allergic Rhinitis Patients. Biomedicines.

